# In vitro and in vivo acaricidal properties of orally delivered ivermectin against the blacklegged tick, *Ixodes scapularis*

**DOI:** 10.1186/s13071-026-07380-7

**Published:** 2026-04-08

**Authors:** Jolieke G. van Oosterwijk, Luciana Richer, Laura K. Beimfohr-Griffing, Andrew Y. Li

**Affiliations:** 1 US Biologic, Inc., 20 Dudley, Suite 900, Memphis, TN 38103 USA; 2 USDA-ARS Invasive Insect Biocontrol and Behavior Laboratory, 10300 Baltimore Avenue, Beltsville, MD 20705 USA

**Keywords:** *Ixodes scapularis*, Ivermectin, Acaricide, White-footed mouse, *Peromyscus leucopus*, Capillary feeding, Mouse bait, HPLC, Tick challenge

## Abstract

**Background:**

The lack of effective and affordable new environmental tick control products is one of the major challenges to the existing control strategies against the blacklegged tick (*Ixodes scapularis*), the vector of Lyme disease affecting public health in the United States. Ivermectin is a systemic antiparasitic pesticide that has been used successfully to control biting flies and ticks infesting livestock. Ivermectin-treated corn has also been shown to be effective against adult ticks feeding on deer. The goal of this study was to assess acaricidal properties of orally delivered ivermectin against the blacklegged tick, *Ixodes scapularis*, for the development of a new mouse bait formulation to control immature stages of the blacklegged tick.

**Methods:**

The efficacy of orally delivered ivermectin against *I. scapularis* was evaluated through in vitro capillary feeding tick-feeding experiments and in vivo animal trials using laboratory-bred white-footed mouse, *Peromyscus leucopus*. Capillary feeding of adult females and nymphs with different concentrations (18.8–600 ppb) of ivermectin resolved in rabbit blood were performed to ascertain necessary ivermectin plasma levels to kill adult and nymphal ticks. Mouse baits dosed with two different ivermectin concentrations (24 and 48 ppm) were fed to mice to analyze the pharmacokinetic properties of ivermectin in mouse serum via HPLC analysis. Subsequent tick-challenge trials were conducted to determine the impact of ingested ivermectin mouse bait against larval or nymphal ticks feeding on the mice.

**Results:**

*Ixodes scapularis* females capillary-fed with rabbit blood containing 300 and 600 ppb demonstrated a significantly higher tick mortality starting at 72 h after the start of capillary feeding. Such ivermectin concentrations also significantly reduced blood-feeding of adult females, as determined by reduced fecal production and engorgement scores. Nymphal capillary feeding experiments were unsuccessful as nymphal ticks in both the control and treatment groups died, likely because of desiccation. In the mouse trials, ivermectin reached peak serum concentrations of 650 ppb and 6715 ppb, respectively, at 2 h after consumption of a single treated pellet containing 80 µg (24 ppm) and 160 µg (48 ppm) ivermectin, respectively. Ivermectin was rapidly depleted from mouse blood with a half-life of < 6 h. Mouse trials showed that ivermectin activity was most effective in controlling larval and nymphal feeding when mice consumed ivermectin-treated bait 24 h before or after tick challenge, with the best results observed in mice fed 48 ppm ivermectin. When larvae were placed 48 h after mice had consumed ivermectin bait, no difference in feeding was observed compared to control.

**Conclusions:**

Results from in vitro and in vivo experiments demonstrated the oral efficacy of ivermectin against different developmental stages of the blacklegged tick. The acaricidal effects of ivermectin against *I. scapularis* nymphs and larvae feeding on white-footed mice, as observed in the mouse trials, may be considered preliminary, and further laboratory and field studies are necessary to validate the utility of an ivermectin-based mouse bait formulation for controlling of immature *I. scapularis* ticks feeding on mice. To our knowledge, this is the first study to show the oral efficacy and PK analysis of ivermectin in mice. This study found that, although efficacious against tick feeding in the first 24 h, ivermectin has a very short half-life in mice and therefore has a short therapeutic window. This study provides important information for the development of mouse bait to control ticks and tick-borne diseases.

**Graphical abstract:**

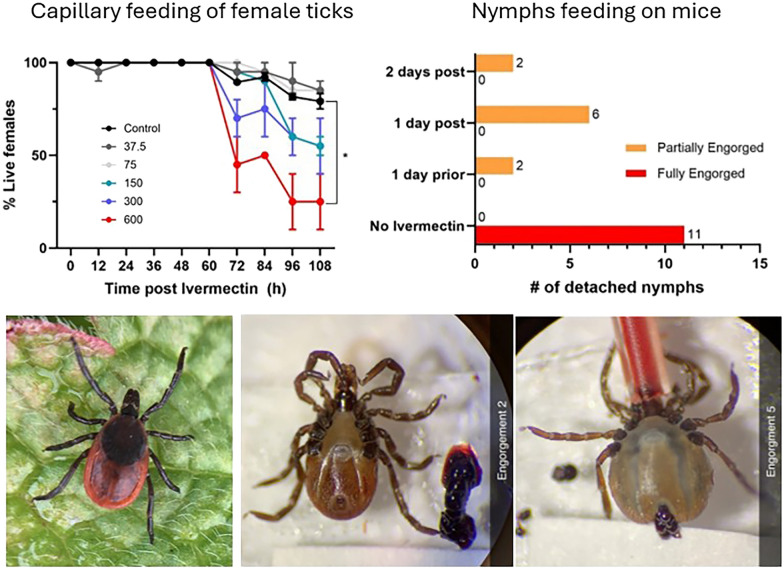

## Background

The US is facing a rapid range expansion of major tick species of medical and veterinary importance, indicating an elevated risk of tick-borne diseases [1–3]. The blacklegged tick, *Ixodes scapularis*, is responsible for transmitting at least seven human pathogens, including *Borrelia burgdorferi,* the causative agent of Lyme disease (LD) [4]. Lyme disease, transmitted only by *I. scapularis*, accounts for > 80% of reported tickborne diseases, making it the most important vector-borne disease affecting humans in the US [5]. In an earlier study by the DHHS Tick-Borne Disease Working Group, Lyme disease, by itself, is estimated to be a $50B—$100B challenge to the US healthcare system [6].

Various personal protection measures, such as repellents, protective clothing, and tick control products, have been studied in recent decades to reduce the risk of tick bites [7, 8]. Chemical acaricides can be effective when applied as sprays and granules to localized tick habitats (wooded edges of suburban home sites) [9–12], but safety and environmental concerns limit regular use of acaricides for tick control. Host-targeted tick control products, including the “4-Poster” deer bait and treatment station and rodent bait boxes, have been successfully used to control ticks on white-tailed deer and white-footed mice [13–18].

The white-footed mouse, *Peromyscus leucopus*, is an important host for immature stages of *I. scapularis* and the reservoir host for *B. burgdorferi* and other tick-borne pathogens. Current mouse-targeted tick control products on the market include mouse bait boxes containing passively applied topical acaricide fipronil and tick tubes with permethrin-treated cotton as mouse nest material [15, 18, 19]. In recent years, oral bait formulations containing systemic acaricides, such as fipronil or Fluralaner, have been developed and evaluated to control immature *I. scapularis* ticks feeding on white-footed mice and to break the life cycle of *I. scapularis* [20–22]. Fluralaner is one of several isooxazoline compounds that have been successfully developed as chewable flea and tick control products for dogs in the past decade [23]. These chewable formulations demonstrated both rapid kill and long-lasting efficacy against infesting ticks after a single oral administration to dogs [24]. It has also been demonstrated that oral administration of sarolaner or afoxolaner to dogs can prevent transmissions of *B. burgdorferi* from infected *I. scapularis* ticks [25, 26]. The recent evaluations of mouse baits containing fipronil or fluralaner represent progress toward repurposing existing acaricides successfully used in veterinary ectoparasite control products for public health use [27, 28].

Ivermectin is an antiparasitic drug that has been used as a veterinary medicine to control biting flies and ticks infesting livestock in recent decades [29–33]. Systemic treatment of white-tailed deer with ivermectin-medicated bait has been shown to be effective in suppressing populations of *Amblyomma americanum* and *I. scapularis* in field studies [34–36]. To our knowledge, no research has been published describing the use of ivermectin in mouse bait formulations to control immature *I. scapularis* feeding on white-footed mice. We conducted a study between 2019 and 2020 to assess the acaricidal properties of orally administered ivermectin against the blacklegged tick, *I. scapularis*, using both in vitro and in vivo laboratory experiments.

## Methods

### Animals

Adult females and nymphs of *I. scapularis* used for capillary feeding experiments were purchased from the Oklahoma State University National Tick Research and Education Resource (Stillwater, OK). *Ixodes scapularis* nymphs and larvae used for mouse infestation were obtained from the CDC National Center for Emerging and Zoonotic Infectious Diseases Division of Vector-Borne Diseases, Rickettsial Zoonoses Branch (Atlanta, GA). Ticks were maintained at 22 ± 2 °C with a relative humidity (R.H.) of 95 ± 2% under a 12:12 h (L:D) photoperiod before use in experiments.

Forty-one white-footed mice, *P. leucopus*, were obtained from the *Peromyscus* Genetic Stock Center at the University of South Carolina (Columbia, SC). Mice were maintained at the animal facility of TriMetis Life Sciences (City, State). Laboratory maintenance of mice and mouse experiments followed the animal use protocol (IACUC 19-0081) approved by the Institutional Animal Care and Use Committee (IACUC) of the University of Tennessee Health Center (UTHSC). During challenge, mice were maintained individually in microisolator caging, with half the cage positioned on a heating pad, allowing the mice to keep warm and move away from the heating source when desired. Mice always had access to water and feed ad libitum unless stated otherwise, and health checks were performed daily.

### Tick capillary feeding experiments

Capillary feeding experiments were performed at the USDA, ARS, Invasive Insect Biocontrol and Behavior Laboratory, Beltsville, Maryland, USA. Technical ivermectin (96.3% a.i., Chem Service Inc., West Chester, PA, USA) was dissolved in dimethyl sulfoxide (DMSO) (Sigma-Aldrich, St. Louis, MO, USA), which was further diluted in purified water to generate stock dilutions containing 0.625–10 ppm ivermectin and 1% DMSO. The stock solutions were further diluted in deiminated rabbit blood (Hemostat Laboratories, Dixon, CA) to the desired test concentration of ivermectin in rabbit blood: 18.75, 37.5, 75, 150, 300, and 600 ppb with 0.03% DMSO. The control blood contained only 0.03% DMSO.

In vitro blood feeding of adult females and nymphs of *I. scapularis* was achieved using 5-µl and 2-µl MicroCaps® capillary tubes (Drummond Scientific Company, Broomall, PA). Each ivermectin concentration included 10 females or nymphs evenly positioned in two standard 9-cm-diameter petri dishes (5 ticks / dish). After each tick had been attached with the ventral side up to the petri dish using double-sided tape, a capillary tube containing blood containing a known concentration of ivermectin was placed over the tick's mouthparts (hypostome and palps). The distal end of each capillary tube was placed on a strip of utility wax to secure the capillary tube. After closing the petri dish with a lid, the petri dish was placed in an incubator and maintained at 85–99% R.H. and 36 °C.

**Fig. 1:**
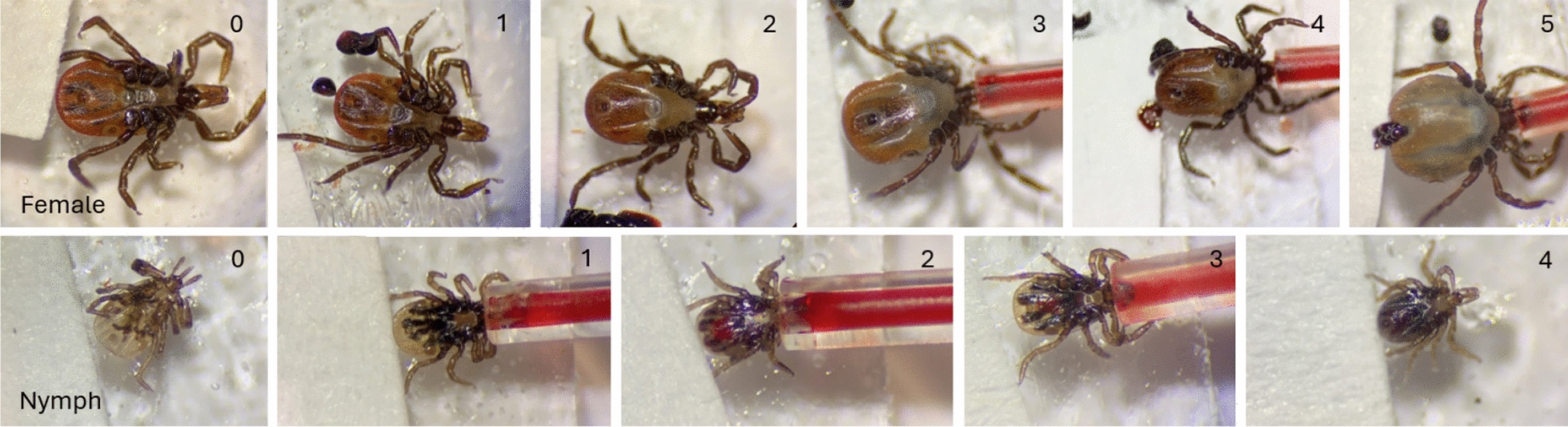
Observed changes associated with capillary feeding of blood in females (top panel) and nymphs (lower panel) of *I. scapularis*. The score, ranging from 0 to 5, assigned to each image represents an estimated stage of engorgement

Capillary tubes were changed twice each with fresh blood with the same ivermectin-blood mixture for ticks in each treatment group. Blood consumption and tick mortality were documented at each change of the capillary tube. Mortality status was categorized as “alive,” “moribund,” or “dead. Ticks were rated based on engorgement level (0–5), with 0 being flat/unfed and five being fully engorged, as documented using a Leica S6D compound microscope equipped with a digital Leica camera (Flexacam C1) (Deerfield, IL, USA; Fig. 1). Fecal pellets were counted and used as an indicator of how well ticks were or were not feeding. Ticks were fed for 4–5 consecutive days, then removed from the petri dish and placed into labeled plastic snap-cap vials with holes poked in the lids. The snap cap vials were then placed into a room temperature humidor. Ticks were monitored for mortality up to 11 days post-treatment. The capillary feeding experiments were replicated three times.

### Mouse trials

*Pharmacokinetic study*: To determine the pharmacokinetic properties in *P. leucopus* after oral administration, feed pellets were treated with ivermectin (I8898, Millipore Sigma, Burlington, MA). Pellets containing either 24 ppm (80 µg/pellet) or 48 ppm (160 µg/pellet) ivermectin were created in the US Biologic laboratory. Mice were housed individually and fasted for 8 h prior to ivermectin administration. Mice had access to water ad libitum during the fasting period and were closely monitored. Each mouse was presented with one pellet of either 24 ppm or 48 ppm ivermectin, and the time was recorded after full consumption of the pellet. Two mice were killed at 2, 3, 4, 5, 6, 12, and 24 h post-administration, at which point blood was collected in non-heparinized tubes via a terminal cardiac bleed. One mouse was used to collect baseline blood. As ivermectin is metabolized by the liver and can be detected in the bloodstream, HPLC analysis was performed at the University of Memphis to detect plasma concentrations in the mice.

Detection of plasma ivermectin concentrations was performed using the previously described fluorescence HPLC method [37, 38]. In short, four parts acetonitrile and one part ddH_2_O were added to four parts of serum and mixed for 30 min. After 5 min of centrifugation at 2000 g, the supernatant was transferred into a clean tube, and the solid phase was derived by drying using nitrogen flow. For derivatization, the residue was dissolved in N-methylimidazole/acetonitrile (1:2 v/v). Samples were incubated with trifluoracetic acid/acetonitrile (1:2 v/v) and incubated for 10 min before injection into the chromatograph using a LiChrospher 100 RP-10 (125 mm × 4 mm) (Sigma) column. Ivermectin was detected using a 360-nm excitation wavelength and 475-nm emission wavelength.

*Tick challenge experiments:* To establish the acaricidal activity of ivermectin against *I. scapularis* ticks, 12 *P. leucopus* mice were used to determine the impact of ivermectin on nymphal and larval ticks in relation to the timing of mouse ingestion of ivermectin pellets. The mice were divided into four treatment groups with three mice in each group: (1) three mice were subjected to a tick challenge without ivermectin exposure, (2) three mice received ivermectin 1 day prior to tick challenge, (3) three mice received ivermectin 1 day post ivermectin, and (4) three mice received ivermectin 2 days post-ivermectin. All mice in a treatment group had 24 h to consume ivermectin-treated pellets, after which their pellets were replaced with normal food. Mice had access to food and water ad libitum. Mice were anesthetized during the challenges and challenged with 8 nymphs and 30 larvae, according to the methods described in Bouchard et al. [39]. During tick challenges, mice were housed separately in FIC microisolator caging with wire bottoms for 8 days. Detached ticks were collected from the bottoms of the cages and inspected for engorgement status. Over the course of 8 days, cages were inspected twice a day for detached ticks and to perform health checks.

### Data analysis

**Fig. 2:**
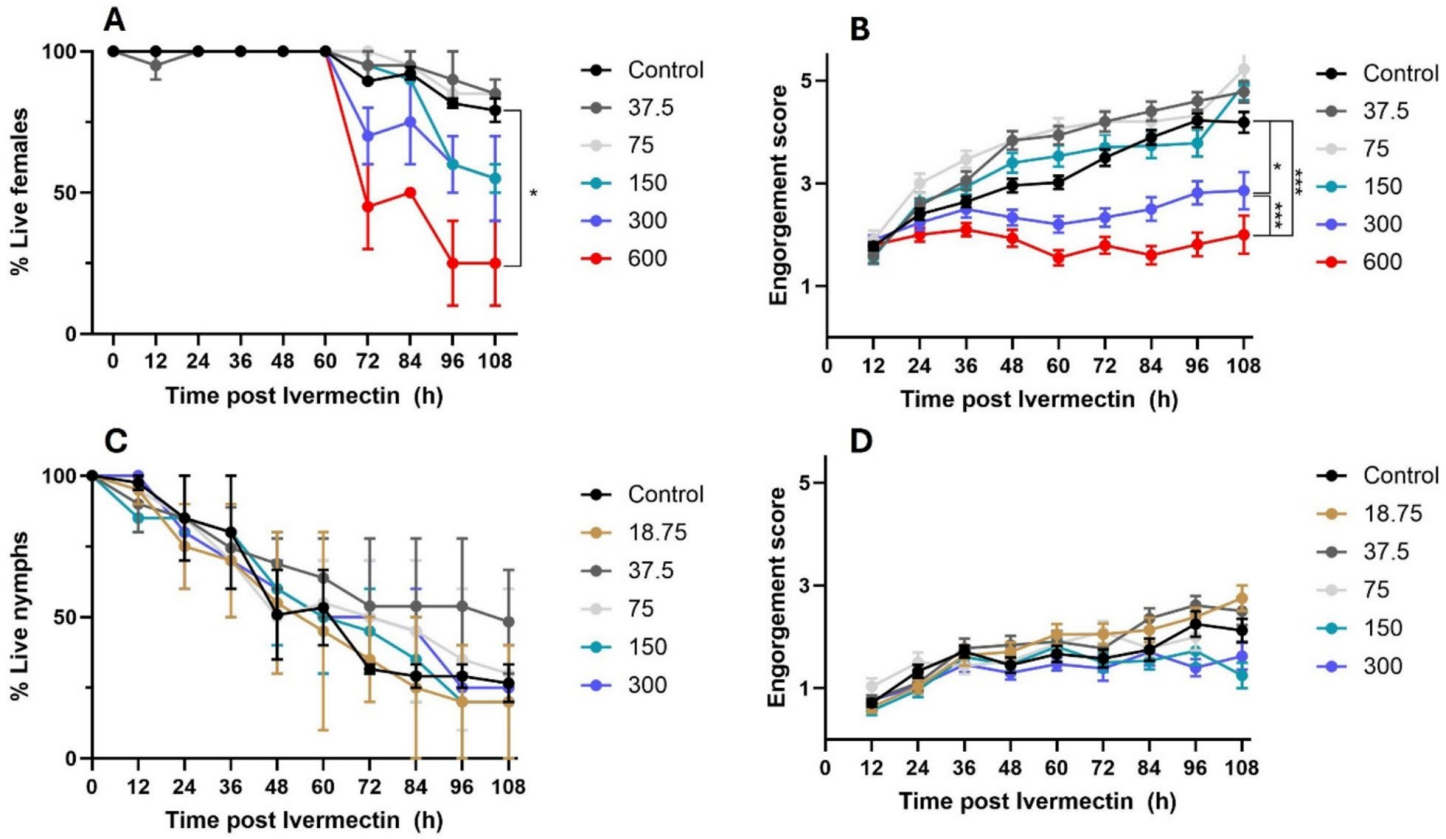
Effects of ivermectin ingested with blood through capillary feeding on the survival (**A**, **C**) and engorgement (**B**, **D**) of adult females and nymphs of *Ixodes scapularis*. Data points on the graph represent the mean and standard deviations. Lines of different colors represent different concentrations (ppb) of ivermection in blood. *, ***Significant difference between means at *p* < 0.05 and *p* < 0.0005 level, respectively

**Fig. 3:**
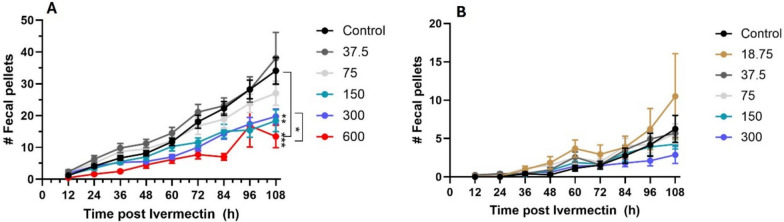
Effects of ivermectin ingested with blood through capillary feeding on tick feeding, as measures by fecal pellet production, in adult females (**A**) and nymphs (**B**) of *Ixodes scapularis*. Data points on the graph represent the mean and standard deviations. Lines of different colors represent different concentrations (ppb) of ivermection in blood. *, **, ***Significant difference between means at *p* < 0.05, *p* < 0.005, and *p* < 0.0005 level, respectively

Figures were created using GraphPad Prism Software (San Diego, CA, USA). The same software was used to compare capillary feeding parameters, including percentage survival, engorgement score, and amount of fecal production between treatment groups (unpaired two-tailed t-test), as presented in Figs. 2 and 3.

## Results

### Ivermectin ingested through capillary tube killed female ticks and reduced tick blood-feeding

Nymphal and female adult *I. scapularis* ticks were exposed to increasing ivermectin concentrations in capillary tubes over the course of 4–5 days. Thirty ticks from each life stage were exposed to each concentration over three trials. Adult *I. scapularis* females showed significantly higher mortality at 150 ppb and 600 ppb of ivermectin than those at lower ivermectin concentrations or the untreated control (Fig. 2A, *p* < 0.05, independent two-tailed t-test). There was a clear concentration-dependent mortality response in female ticks in response to different concentrations of ivermectin in the blood. The negative impact of ivermectin on tick blood-feeding was similarly demonstrated in female ticks feeding on rabbit sera, with 300 ppb or 600 ppb of ivermectin showing significantly lower levels of engorgement (*p* > 0.05 and *p* < 0.0005, respectively, independent two-tailed t-test; Fig. 2B).

In contrast, no concentration-dependent effect of ivermectin on mortality or engorgement score was observed in nymphal *I. scapularis* ticks, which could in part be due to the reduced viability of these nymphal ticks throughout the experiment (Fig. 2C, D). Examination of tick fecal pellet production during capillary blood-feeding showed that adult female *I. scapularis* ticks had significantly fewer fecal pellets (*p* < 0.05 and *p* < 0.0005 in ticks fed on blood with 300 ppb and 600 ppb ivermectin, respectively), indicating that ivermectin inhibits tick feeding behavior (Fig. 3A). No significant differences were found among different treatment groups in nymphal fecal pellet production (Fig. 3B).

### Steep decline in ivermectin concentration in mouse serum after ingestion

HPLC analysis of serum samples prepared from blood plasma collected at different times after mice were given a single ivermectin-treated pellet containing either 24 ppm or 48 ppm of ivermectin and standard non-compartmental pharmacokinetic analysis were performed to estimate ivermectin bioavailability in mice after oral consumption of ivermectin.

**Fig. 4:**
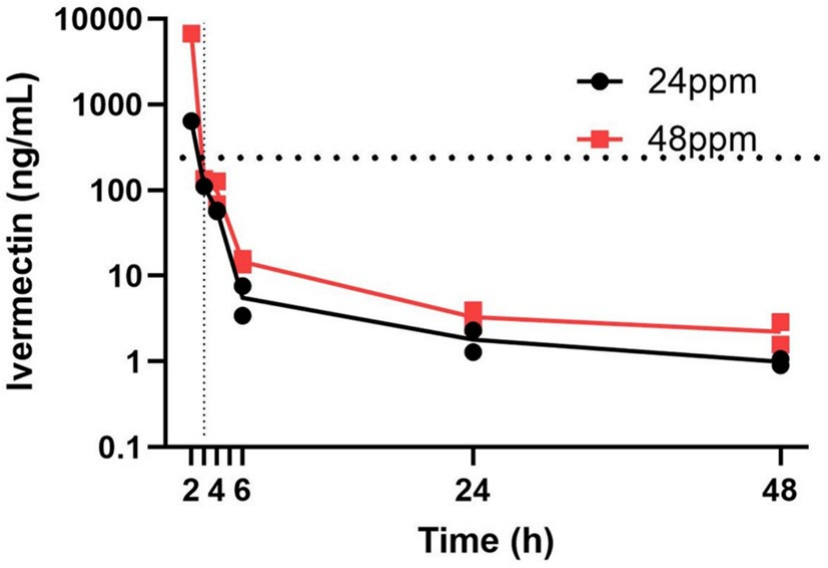
Change in serum ivermectin concentration (ng/ml or ppb) at different times (hours) after mice ingested a single diet pellet containing 24 ppm (80 µg) or 48 ppm (160 µg) of ivermection. Each data point represents one mouse

The peak concentration (Cmax) of ivermectin observed in mouse blood plasma was during the first collection time point, at 2 h post-consumption, after which the serum ivermectin concentration declined rapidly. For the high dose, 48 ppm delivered orally, the initial ivermectin concentration measured was 6715 ng/ml at 2 h post-ingestion (AUC 3881, 95% CI 3780–3982), and for the low dose, 24 ppm delivered orally, the initial ivermectin concentration was 650 ng/ml at 2 h post-ingestion (AUC 622.8, 95% CI 566.3–679.3) (Fig. 4). The relative bioavailability (Frel) of doubling the dose of ivermectin orally from 24 to 48 ppm in this study led to a 1.7 increase in the total bioavailability over time as calculated by the AUC. The most rapid decline was observed during the first 6 h, at which point mice ingesting 24 ppm ivermectin had an average blood plasma level of 6 ng/ml, and mice ingesting 48 ppm ivermectin had an average blood plasma level of 14 ng/ml. At 24 h, the serum concentration of ivermectin ranged from 2 to 4 ng/ml and continued to decline to around 1–3 ng/ml (Fig. 4).

### Ivermectin orally ingested by mice inhibited feeding of both larval and nymphal ticks

**Fig. 5:**
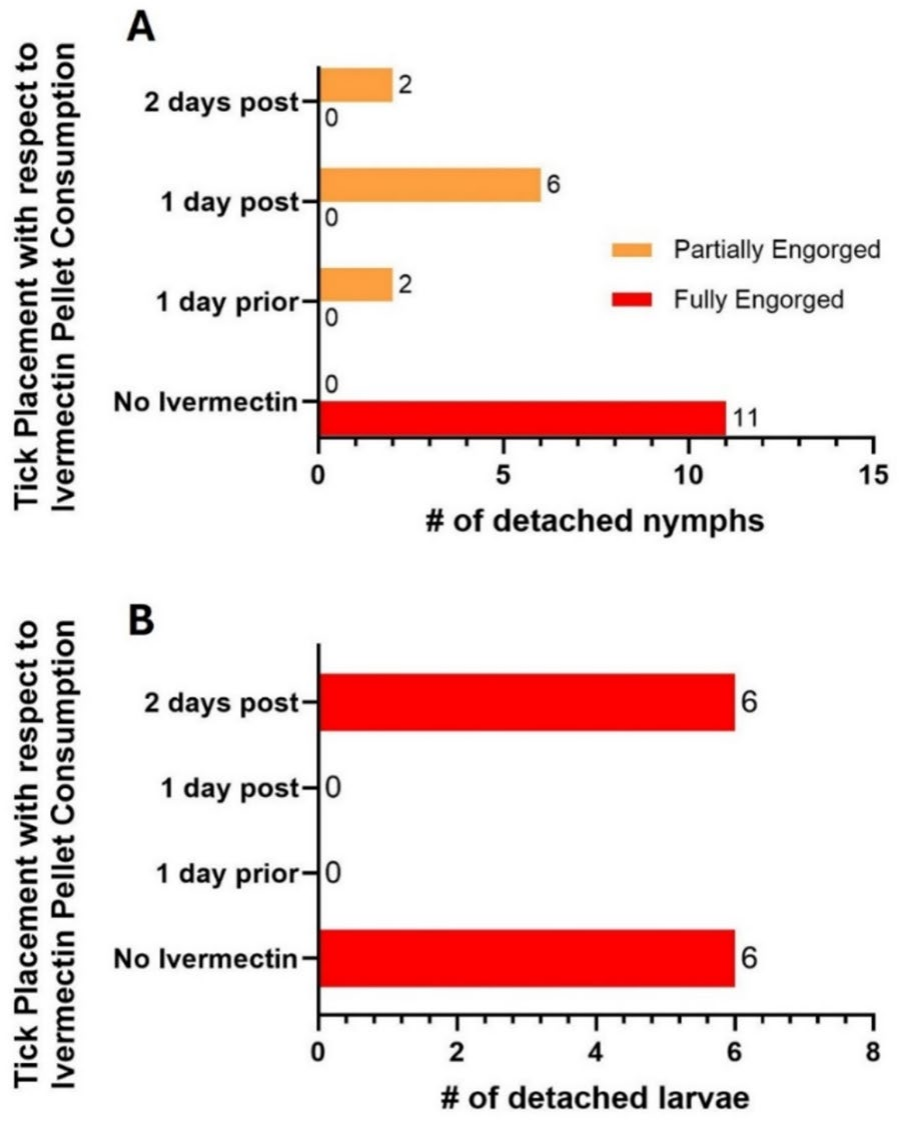
Results of the in vivo mouse trial on the effect of ingesting ivermectin-treated pellets (48 ppm) on the blood-feeding success of nymphs (**A**) and larvae (**B**) of *Ixodes scapularis* in relation to timing of tick infestation and mouse access to ivermectin-treated pellets. *Two days post*: tick infestation occurred 2 days after mice had fed on ivermectin-treated pellets; *1 day post*: tick infestation occurred 1 day after mice had fed on ivermectin-treated pellets; *1 day prior*: tick infestation occurred 1 day before mice had fed on ivermectin-treated pellets; *No ivermectin*: tick infestation on mice feeding on regular untreated pellets (untreated control)

Figure 5 summarizes the results of in vivo tick challenge experiments. Mice that had been started on ivermectin pellets 1 day prior to and 1 day after to tick infestation (Fig. 5) showed a 100% inhibition of larval and 45.5% to 81.8% inhibition of nymphal feeding, as illustrated by the reduction of fed nymphs and larvae observed at the bottom of the wire cages after placement (Fig. 5A, B). A similar level (81.8%) of inhibition on nymphal feeding was observed when nymphs were placed on mice 2 days after the mice had been given ivermectin-treated pellets (Fig. 5A). However, no inhibition on larval feeding was observed when larvae were placed on mice 2 days after the mice had been were given ivermectin-treated pellets compared with the untreated control group (Fig. 5B).

## Discussion

Previous studies have demonstrated that ivermectin-treated corn fed to white-tailed deer is effective against *I. scapularis* ticks feeding on them [33–36]. To determine whether the same results could be obtained using ivermectin-treated bait fed to mice, we performed capillary feeding trials, feeding ticks ivermectin concentration in blood, then PK trials in mice after controlled ivermectin bait consumption, and finally we performed tick challenge trials in mice. Through capillary feeding experiments, we determined the minimal lethal concentration of ivermectin to *I. scapularis* ticks. Ticks were fed continuously for 4–5 days with blood meals containing five increasing concentrations of ivermectin delivered via capillary tubes. The minimal lethal ivermectin concentration was determined to be 300 ppb in adult females. Unfortunately, in the current study, the minimal lethal ivermectin concentration for nymphs could not be determined, as the nymphs did not do well when fed through capillary tubes. Nymphs in all treatment groups died similarly over the course of 4 ½ days, probably because of repetitive handling. The discrepancy between the lethal concentrations observed in field trials and those in the capillary feeding trials is likely due to the nature of the capillary feeding trials, as feeding was repeatedly interrupted while capillaries were refreshed.

Ivermectin pharmacokinetics are well characterized for many larger animals, for both topical and oral administration. In dogs, oral formulations of ivermectin are commonly used to prevent heartworm and endoparasites, with a monthly dosage providing adequate therapeutic ivermectin plasma concentrations [40]. Subcutaneous and oral ivermectin both lead to higher plasma concentrations in cattle than in sheep and goats. Oral ivermectin led to the highest bioavailability in cattle with a maximum serum concentration at an average of 31.1 h and an average elimination time of 33.8 days, and the lowest bioavailability in goats at an average of 11.5 h and an average plasma elimination time of 16.6 days [41].

This study shows that ivermectin metabolism in mice is markedly faster than in large animals, such as the white-tailed deer [35]. In mice, ingestion of a single oral dose of ivermectin formulated in a pellet leads to maximum serum ivermectin concentrations at 2 h post consumption. Maximum serum concentrations for high-dose ivermectin were higher than for low-dose ivermectin: a low dose (80 µg, 24 ppm) led to a maximum serum concentration of 650 ppb, and high dose oral ivermectin (160 µg, 48 ppm) led to a maximum serum concentration of 6715 ppb (Fig. 4). The ivermectin serum concentration decreased rapidly after reaching Tmax, dropping to 10 ± 5 ppb in only 6 h after pellet ingestion.

Using challenge studies, we confirmed that the ivermectin concentration in mouse blood was effective in impeding blood-feeding by immature *I. scapularis* ticks feeding on mice. Notably, any live nymphs collected from the treatment groups were partially fed, while all nymphs collected from the control group were fully fed. In accordance with the pharmacokinetic analysis, the challenge data show that larval feeding is fully inhibited when larvae are placed 1 day before or 1 day after ivermectin consumption by the mice, as they will be actively feeding during high plasma concentrations of ivermectin. The lack of effect when placing the larvae 2 days after ivermectin treatment is expected to be because plasma concentrations of ivermectin have dropped too low by the time the larvae have fully attached and started feeding.

Notably, the curve for the metabolism of ivermectin in mice, with a steep increase to maximum plasma concentrations and then rapid clearance, is similar to the curve for the metabolism observed by Myers et al. [40] in ruminants. However, in mice, the time during which maximum serum concentration and subsequent clearance occur is markedly shorter than that observed in either dogs or ruminants [40–42]. A study by Pelletier et al. [20] found that fluralaner, an acaricide used in tick control, showed similar discrepancies in PK profiles when administered orally to mice.

Fluralaner is used in dogs as an oral treatment in tick prevention, with efficacy against ticks up to 2 months post-treatment. However, when used as an oral bait in mice, effective Fluralaner plasma concentrations were only detected at 2 days after bait administration [20]. The consist rapid clearance of these acaricides in mice could be due to a several factors. The increased metabolism in mice compared to larger animals could play a role in faster clearance. Similarly, as these acaricides are cleared via the hepatobiliary pathway, Pelletier et al. [20] posed that the increased hepatic clearance in mice could play a role. In addition, metabolism and hepatic clearance can differ between species. Notably, the CYP enzymes and drug transporters in the liver are not always directly comparable across species [42]. Ivermectin is a substrate and inhibitor for cytochrome P450 [43], which shows different abundances and enzyme activities depending on the species [42, 44]. Therefore, when developing oral baits containing acaricides, performing pharmacokinetic and efficacy studies is crucial, as results cannot be directly extrapolated from one species to another.

As part of ongoing research efforts to re-purpose older acaricides for public health use, we selected ivermectin for evaluation considering its previous use to control ticks in white-tailed deer and cattle [31, 33–36]. Although we demonstrated ivermectin's efficacy as a systemic acaricide against *I. scapularis* ticks in laboratory studies, results from our study revealed certain disadvantages that may render ivermectin a less desirable active ingredient for use in mouse baits to control immature *I. scapularies* ticks feeding on white-footed mice.

Results from our study suggest that ivermectin is efficacious against immature ticks feeding on mice for a few hours after the mouse has ingested the medicated bait, resulting in mortality of the attached ticks. This tick-killing effect may be significant, as it could interrupt *B. burgdorferi* transmission during this time. The rapid decline in serum ivermectin concentration after bait ingestion by mice requires more frequent application of the mouse bait formulation, potentially increasing the risk of ivermectin resistance in field populations of *I. scapularis*. On the other hand, a quick depletion of ivermectin from mouse blood might be better for lowering resistance risks than sustained sub-efficacious levels. Although ivermectin resistance has not been reported in *I. scapularis*, it has been well documented in the cattle tick and the brown dog tick [45, 46]. In addition, it should be noted that ivermectin has an unfavorable oral toxicity profile (LD_50_ = 21 mg/kg) to mice compared with other acaricides, such as for fipronil (LD_50_ = 95 mg/kg) [47–49]. Many factors may affect the field success of a pest control product. The current study represents an initial laboratory assessment of the acaricidal properties of orally delivered ivermectin against the blacklegged tick. Based on our findings, we conclude that ivermectin is not a favorable candidate for field application.

## Conclusions

This study demonstrated a critical approach to evaluating an existing acaricidal compound for the development of new medicated mouse bait formulations for public health use. The observations of stark differences in the pharmacokinetic behavior of ivermectin in mice compared to other species may propel other researchers in the field to consider the importance of timing of administration and the therapeutic window in testing and developing new acaricidal compounds or re-purposing existing pesticide compounds for public health use.

## Data Availability

Data supporting the conclusions of this article are included in the article.
